# Photosynthetic Efficiency and Glyco-Metabolism Changes in Artificial Triploid Loquats Contribute to Heterosis Manifestation

**DOI:** 10.3390/ijms231911337

**Published:** 2022-09-26

**Authors:** Lingli Wang, Meiyan Tu, Jing Li, Shuxia Sun, Haiyan Song, Zihong Xu, Dong Chen, Guolu Liang

**Affiliations:** 1 Horticultural Research Institute, Sichuan Academy of Agricultural Sciences, Chengdu 610066, China; 2 College of Horticulture and Landscape Architecture, Southwest University, Tiansheng Road 2, Chongqing 400715, China

**Keywords:** triploid loquat, heterosis, photosynthetic efficiency, glyco-metabolism, transcriptome sequencing

## Abstract

Previous studies indicated that extensive genetic variations could be generated due to polyploidy, which is considered to be closely associated with the manifestation of polyploid heterosis. Our previous studies confirmed that triploid loquats demonstrated significant heterosis, other than the ploidy effect, but the underlying mechanisms are largely unknown. This study aimed to overcome the narrow genetic distance of loquats, increase the genetic variation level of triploid loquats, and systematically illuminate the heterosis mechanisms of triploid loquats derived from two cross combinations. Here, inter-simple sequence repeats (ISSRs) and simple sequence repeats (SSRs) were adopted for evaluating the genetic diversity, and transcriptome sequencing (RNA-Seq) was performed to investigate gene expression as well as pathway changes in the triploids. We found that extensive genetic variations were produced during the formation of triploid loquats. The polymorphism ratios of ISSRs and SSRs were 43.75% and 19.32%, respectively, and almost all their markers had a PIC value higher than 0.5, suggesting that both ISSRs and SSRs could work well in loquat assisted breeding. Furthermore, our results revealed that by broadening the genetic distance between the parents, genetic variations in triploids could be promoted. Additionally, RNA-Seq results suggested that numerous genes differentially expressed between the triploids and parents were screened out. Moreover, KEGG analyses revealed that “photosynthetic efficiency” and “glyco-metabolism” were significantly changed in triploid loquats compared with the parents, which was consistent with the results of physiological indicator analyses, leaf micro-structure observations, and qRT-PCR validation. Collectively, our results suggested that extensive genetic variations occurred in the triploids and that the changes in the “photosynthetic efficiency” as well as “glyco-metabolism” of triploids might have further resulted in heterosis manifestation in the triploid loquats.

## 1. Introduction

Polyploidy or whole-genome duplication (WGD) is characteristic of an organism whose genome contains more than two complete sets of chromosomes, which is common in the plant kingdom [1]. Studies revealed that the genomes of modern plants have undergone multiple rounds of past polyploidization events, which is considered as a driving force in the process of plant evolution [2,3,4]. For angiosperms, the incidence of polyploidy recorded in the literature is variable, ranging from 30% to 70% [5,6]. This ubiquitous phenomenon has attracted biologists for almost a century due to the fact that plant polyploidization is often accompanied with the appearance of some new traits, increased vigor, etc., compared with their diploid relatives, such as a higher resistance to biotic and abiotic stresses, altered flowering time, and increased organ size, which make polyploids better survive in some extreme environments [7,8,9]. These useful features greatly attracted breeding scientists and led them to induce polyploidy or improve plant cultivars using natural polyploidy [3].

However, with an increased genome dosage, polyploidization often triggers extensive genomic instabilities, causing genetic variations such as genomic rearrangements (genomic exchange and gene loss) [2,10,11]. It was reported that the more closely genomes are related to polyploidy, the more likely it is for them to match homoeologous chromosomes, leading to chromosomal exchanges between the two genomes during meiosis [12]. In the meantime, genome duplication often leads to a larger number of duplicate genes. To maintain the functions well, these duplicate genes undergo a selective loss of some repetitive genes or sequence eliminations [13,14]. For instance, it was found in all studies on *Brassica* and *Tragopogon* that the elimination of genome sequences could occur during polyploid formation [15,16], which was precisely because genome duplication could generate the vast majority of duplicate genes and redundant sequences; therefore, understanding the genetic variations after polyploid formation is critical for us to elucidate the process of polyploidization.

Heterosis, or hybrid vigor, was firstly put forward by Darwin in 1876 and refers to the superior growth potential of hybrids compared with their parents [17]. Its application in agricultural industry has brought about a great production improvement for many major crops, such as maize [18], rice [19], soybean [20], etc. It is estimated that crop yields are improved by 15–50% through heterosis [21]. Regrettably, the underlying mechanisms of heterosis still remain to be elucidated. Previously, three genetic-based hypotheses were raised to explain the heterosis phenomenon, namely, the dominance, over-dominance, and epistasis hypotheses [22,23,24]. Although these hypotheses could be used to interpret the heterosis phenomenon to some extent, none of them could be used to fully illuminate its underlying mechanisms. Theoretically, phenotypes might be controlled via gene expression; thus, two gene expression models were proposed to explain the heterosis phenomenon, including the additive expression and non-additive gene expression patterns [25]. Based on this, it was found in some studies that heterosis was highly associated with the additive expression pattern and that the transgressive characters of hybrids could be produced through the non-additive gene pattern [26,27]. However, how is this phenomenon caused, since no new genes are created in hybrids? Previous studies indicated that the loss, mutation, and divergence of duplicate genes in polyploids might play a critical role in regulating the expression of those genes [28,29]. Therefore, investigating genetic variations after the formation of a polyploid would be helpful for elucidating the heterosis mechanisms.

Loquats (*Eriobotrya japonica* (Thunb.) L.) belong to subtribe *Pyrinae*, *Rosaceae* family, which is indigenous to southeast China and is currently cultivated worldwide [30]. Loquats are favored by many people due to their great taste and rich nutritional value [31,32]. In addition, it is found in traditional Chinese medicine that both loquat fruits and leaves have medicinal applications [33,34]. However, currently, all the loquat fruits sold in the market are diploids that generally carry four–seven large seeds, and this has greatly affected the fruit edible rate [35]. To our surprise, it was found in our previous studies on triploid loquats that triploid loquats were not only seedless, but they also exhibited greater growth vigor than diploid and tetraploid relatives despite the existence of the ploidy effect [36,37]. How can this happen without the ploidy effect? One possible explanation is that significantly more genetic variations may occur in odd ploidy plants than in even ploidy plants [38]. So, by elucidating the genetic variations in triploid loquats after their formation, more details could be provided for elucidating the above phenomenon (heterosis) in triploid loquats as well as triploid loquat breeding. However, it is quite hard for us to carry out relative research on the genetic variations in triploid loquats due to the fact that the origin and evolution of loquats are still unclear, which makes it impossible for us to conduct a study using natural triploid loquats, whereas cross-breeding provides us with an effective solution to this problem since tetraploid loquats have limited fertility, based on reported studies, and the egg cells of tetraploid loquats produced via megaspore-mother-cell meiosis contain a certain number of viable diploid egg cells, which could be fertilized by the diploid father [39]. Based on this, Wang (2008) successfully obtained several triploid loquats via cross-breeding (4× × 2×) and analyzed their genetic variations after their formation using the AFLP marker [40]. It was found that similarly to some other polyploids, extensive genetic variations occurred during the formation process of triploid loquats. However, due to the limitations of the obtained triploid loquats, studies on the genomic variations in triploid loquats after their formation remain preliminary, and more triploid loquats still need to be created via hybridization. On the other hand, it was found in studies that cultivated loquats demonstrated a narrow genetic distance, which greatly affected the variation level of triploid loquats formed via hybridization [32].

In the present study, to broaden the genetic distance and obtain more triploid loquats, two Guizhou wild loquats were used to broaden the genetic distance; they acted as father parents for the hybridization with a cultivar tetraploid mother parent to create triploid loquats with a clear genetic pedigree. The genetic variations in the created triploid loquats were systematically analyzed using the ISSR and SSR molecular markers. Meanwhile, to deeply elucidate the heterosis mechanisms of those triploid loquats, transcriptome sequencing (RNA-Seq) was performed. Our research could provide more details for understanding the heterosis mechanisms of triploid loquats and triploid loquat breeding in the future. 

## 2. Results

### 2.1. Ploidy Validation of the Hybrids Obtained with the Two Cross Combinations

In this study, for accuracy, two different methods (chromosome preparation and flow cytometry) were employed for the ploidy validation of the hybrids. According to the results of chromosome preparation, as displayed in Figure S1, there were not only triploid loquats but also some aneuploidy hybrids in both cross combinations of the hybrids, such as chromosome numbers of 45, 50, etc. (Figure S1). Importantly, all the 12 triploids in the two cross combinations were validated to contain the expected chromosome number (2*n* = 51) (Figure S1). Subsequently, the ploidy of the 12 triploid loquats further underwent verification through flow cytometry, and the results showed that the tetraploids demonstrated twice the luminescent value of GC-1/GC-23 and that triploids had 1.5 times the luminescent value of GC-1/GC-23 (Figure S1). Collectively, all the results described above suggested that the 12 hybrids were triploid loquats. 

### 2.2. Leaves of Triploid Loquat Exhibited Significant Heterosis Compared with Diploid and Tetraploid Parents

To further ascertain the growth vigor of the triploid hybrids, five physiological indicators, namely, soluble starch (SS), soluble protein (SP), chlorophyll A (CA), chlorophyll B (CB), and total chlorophyll (TC), were analyzed. Meanwhile, the paraffin section was adopted to observe the leaf micro-structures of the triploids and parents. As is shown in Table S2, almost all the triploid hybrids demonstrated MPH for SS except for A-7 and B-2. For CA, CB, and TC, besides A-4 and B-3, the others also demonstrated MPH. However, among the five physiological indexes, although the SP content in the hybrids did not show obvious MPH, it exhibited a prominent ploidy effect on the triploids (Table S2). Additionally, the results of leaf micro-structures also showed that the leaves of the two diploid parents were thinner than those of the female parent, LQ-1. The hybrids of Triploid-A all demonstrated MPH, and so did the hybrids of Triploid-B (Table S3, Figure S2). Collectively, the results described above suggested that triploids of Triploid-A and Triploid-B demonstrated significant heterosis compared with their parents. 

### 2.3. Screening Suitable ISSR and SSR Markers for the Two Cross Lines

**Figure 1:**
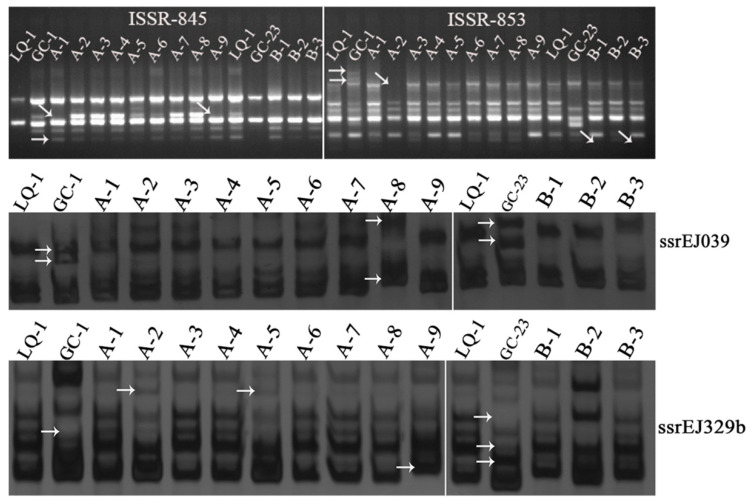
Typical ISSR and SSR profiles for triploid loquats and their parents. ISSR-845 and ISSR-853 were two ISSR markers; ssrEJ039 and ssrEJ329b were two SSR markers; LQ-1 was the tetraploid female parent; GC-1 and GC-23 were two diploid male parents; A-1, A-2, A-3…A-9 were the triploid hybrids of Triploid-A; B-1, B-2, and B-3 were the triploid hybrids of Triploid-B. The arrows indicated the sites of the polymorphic bands.

To obtain the effective primers of ISSR and SSR for the following analyses, the DNA of LQ-1 and GC-1 was adopted for primer screening. For ISSR markers, 100 ISSR primers were detected, and finally, 20 suitable primers were obtained, which could be used for further analyses. For SSRs, all the 20 primers were found to amplify the clear bands. Thus, the 20 ISSR and 20 SSR primers were further used to distinguish the polymorphic bands of the triploid loquats in the two cross lines from those of their parents. The results showed that all the ISSR and SSR primers could be used to screen out clear bands, almost all of which were polymorphic. The examples of typical ISSR and SSR profiles of the triploid loquats and their parents are shown in Figure 1.

### 2.4. Polymorphism Analysis of ISSR and SSR Markers

Totally, 128 loci were screened out from the two cross lines through 20 ISSR primers, of which 1 to 9 per marker had an average of 6.4. Among them, 56 (43.75%) loci were polymorphic sites, of which 0 to 6 per marker had an average of 2.8. The polymorphism ratios of the ISSR markers ranged from 0.00% for ISSR-811, ISSR-858, ISSR-886, and ISSR-892 to 83.33% for ISSR-899. The polymorphic information content (PIC) of these markers ranged from 0 for ISSR-892 to 0.930 for ISSR-874, with an average of 0.733 (Table 1). Based on Botstein et al. (1980), if a PIC value > 0.5, the primer might demonstrate a high discrimination power [41]. Interestingly, in this study, we found that among the 20 ISSR markers, only ISSR-858 and ISSR-892 had a PIC value lower than 0.5, which suggested that almost all of these ISSR markers showed a high discrimination power in analyzing the genetic diversity of loquats.

For SSR analyses, 20 primers obtained from Wu et al. (2015) totally obtained 88 alleles, which was basically consistent with the result (79 alleles) obtained by Wu et al. (2015) [32] (Table 1). The number of alleles per locus ranged from 1 for ssrEJ106 to 6 for ssrEJ046, ssrEJ049, ssrEJ075a, and ssrEJ282 with an average of 4.4. Among all alleles, 17 (19.32%) were polymorphic sites, and the polymorphism ratios of the SSR markers ranged from 0.00% to 60.00%, and ssrEJ039, ssrEJ271, and ssrEJ329b showed the highest polymorphism ratio (60.00%). The Shannon index of the 20 SSR markers ranged from 0.185 (ssrEJ014, ssrEJ037, ssrEJ039, ssrEJ271, and ssrEJ329b) to 0.693 (ssrEJ005 and ssrEJ095b) with an average of 0.302. *H*o, as a measure of marker diversity, ranged from 0.091 to 0.909, with a mean of 0.227, and similar values for *H*e were also calculated (Table 1). Wright’s fixation index (*F*) was calculated for estimating the degree of allelic fixation, and the value of *F* ranged from −0.748 for ssrEJ046, ssrEJ049, and ssrEJ324 to 0.479 for ssrEJ005, with an average of −0.122 (Table 1). Finally, the PIC values of SSR markers were also analyzed as those of the ISSR markers, through which we found that ssrEJ282 had the maximum PIC value (0.872) and ssrEJ106 had the minimum value (0). Only two SSR markers (ssrEJ061 and ssrEJ106) showed a PIC value lower than 0.5 (Table 1). This indicated that almost all of the SSR markers used in this study also exhibited a high discrimination power in analyzing the genetic diversity of loquats.

### 2.5. Genetic Variation Pattern Analyses of Triploid Loquats

**Figure 2:**
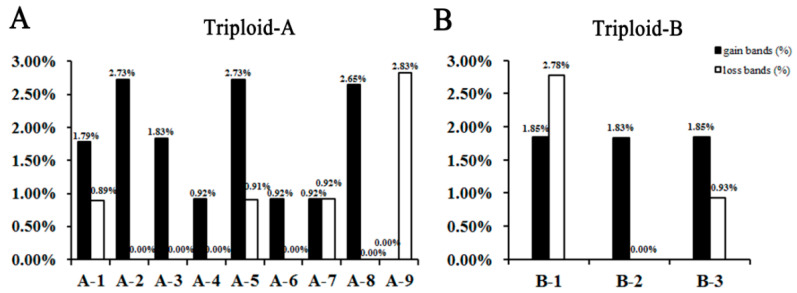
Percentages of “gain bands” and “loss bands” in the Triploid-A lines (**A**) and Triploid-B lines (**B**). A-1, A-2, A-3…A-9 were the triploid hybrids of Triploid-A; B-1, B-2, and B-3 were the triploid hybrids of Triploid-B.

To further ascertain the genetic variation patterns of triploid loquats after their formation, the polymorphic bands of the ISSR markers produced in them were subsequently analyzed. Among these polymorphic bands, two major types of genetic changes were uncovered to be related with triploid variations. As was shown in Figure 2, in Triploid-A, the ratio of the “gain bands” ranged from 0.00% to 2.73%, and the “loss bands” ranged from 0.00% to 2.83% (Figure 2A). Among the hybrids of Triploid-A, only A-9 did not present “gain bands”, and none among A-2, A-3, A-4, A-6, and A-8 presented “loss bands” (Figure 2A). However, in Triploid-B, the “gain bands” of triploid loquats ranged from 1.83% to 1.85%, and the “loss bands” ranged from 0.00% to 2.78% (Figure 2B). The “gain bands” appeared in all the three hybrids of Triploid-B, and only B-2 did not present “loss bands” (Figure 2B). Surprisingly, in both Triploid-A and Triploid-B, the ratios of the “gain bands” were significantly higher than those of the “loss bands” (Figure 2), which indicated that a myriad of genetic variations, such as genetic recombination, chromosomal rearrangement, etc., were produced after the formation of triploid loquats.

### 2.6. Genetic Diversity Analysis

**Figure 3:**
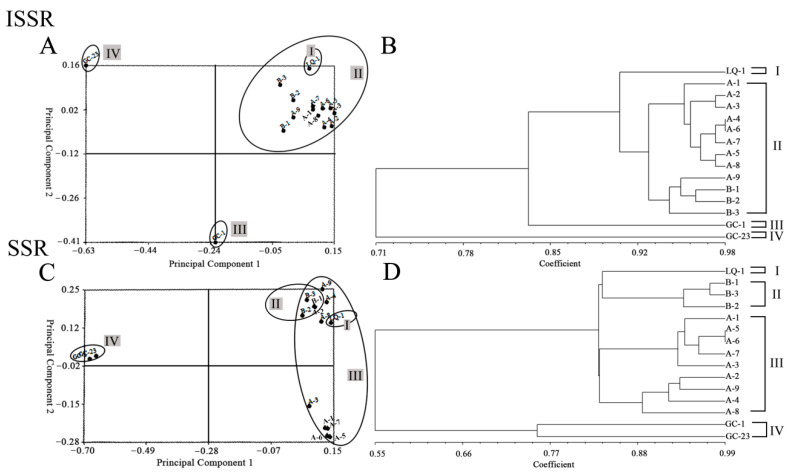
Two-dimensional plot of the principal components analyzed in the two cross lines using SM coefficient (**A**,**C**), and dendrogram of the two cross lines based on ISSR and SSR data as clustered using UPGMA (**B**,**D**); LQ-1 was the tetraploid female parent; GC-1 and GC-23 were two diploid male parents; A-1, A-2, A-3…A-9 were the triploid hybrids of Triploid-A; B-1, B-2, and B-3 were the triploid hybrids of Triploid-B.

As described in the Introduction, elucidating the genetic variations in triploid loquats after their formation could help us to better carry out triploid breeding, which would also be beneficial for uncovering the mechanisms of triploid heterosis. In order to analyze the genetic diversity of the triploid loquats of the two cross lines, two cluster analyses were carried out based on ISSR and SSR markers. In the dendrogram constructed through ISSR, 15 genotypes were clustered into two groups (I + II + III and IV) with the coefficient value of 0.71, and the two wild diploid male parents were not clustered into one group (Figure 3B). However, for the coefficient value of 0.90, all the accessions were clustered into four groups, I (LQ-1), II (hybrids), III (GC-1), and IV (GC-23), and the hybrids could be successfully identified from their parents (Figure 3B). The results also indicated that hybrids from both the two cross lines had a closer relationship with the female parent (LQ-1). On the other hand, we found that ISSR markers could not distinguish the hybrids between Triploid-A and Triploid-B (e.g., A-9 was clustered with B-1 and B-2) or between the two wild male parents (e.g., GC-1 was clustered with the cultivated loquats) (Figure 3B).

For the SSR cluster analysis, the 15 genotypes totally produced two main clusters (I + II + III and IV) for the coefficient value of 0.55 (Figure 3D). The first cluster (IV) contained the two male parents and the wild loquats, and the second cluster (I + II + III) contained the cultivated loquat (LQ-1) and the hybrids (Figure 3D). This suggested that SSR markers could successfully distinguish the wild loquats from the cultivated ones. In addition, for the coefficient value of 0.836, Triploid-B (in II) was successfully separated from Triploid-A (in III) and the tetraploid female parent, LQ-1 (in I) (Figure 3D). These results were consistent with the reality. 

Finally, to further confirm the ISSR and SSR cluster results, PCA analyses were carried out using the similarity matrices of the two molecular markers. For ISSR markers, as it is shown in Figure 3A, the 15 genotypes were distributed into different groups. GC-1 showed a long distance with respect to GC-23, and they were located in groups III and IV, respectively. Triploid-A and Triploid-B (II) showed a narrow distance with respect to the female parent, LQ-1 (I) (Figure 3A). These results were in accordance with the cluster results of ISSR. For SSR markers, a very narrow distance was shown between the two wild male parents, GC-1 and GC-23, which were distributed in group IV (Figure 3C). In addition, B-1, B-2, and B-3 were also gathered together. These were consistent with the cluster results of SSR (Figure 3C). 

### 2.7. Correlation Analysis of ISSR and SSR Markers Using Mantel Test

**Figure 4:**
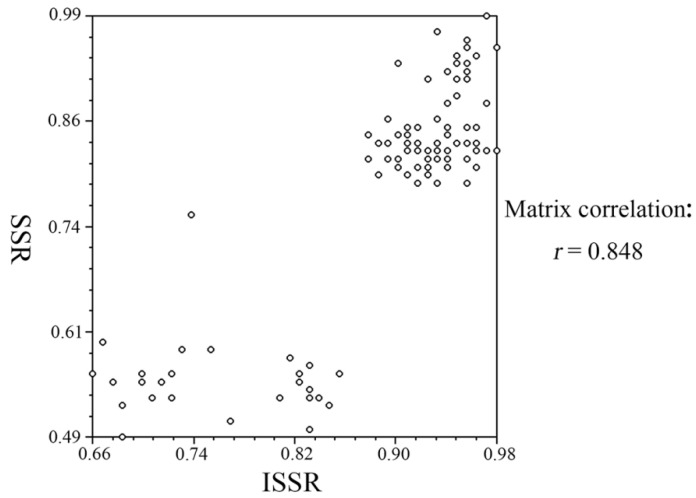
Correlation analysis of the ISSR and SSR markers by using the Mantel test.

Based on the results described above, both ISSR and SSR demonstrated that extensive genetic variations occurred after the formation of triploid loquats. To investigate whether there was some degree of correlation between ISSR and SSR markers as well as the analytical accuracy of ISSR and SSR above, the data of the two markers were further used to perform the Mantel test. Finally, we found that the correlation coefficient between the ISSR and SSR was *r* = 0.848 (*p* < 0.01), which indicated that there was a correlation existing between the results of ISSR and SSR markers, further suggesting that both ISSR and SSR results were reliable (Figure 4).

### 2.8. Transcriptome Analyses of the Parental and Triploid Leaf Tissues

Based on the results obtained above, extensive genetic variations were detected via the ISSR and SSR markers in the hybrids, and since those variations would ultimately alter the gene transcription level, which could reprogram the signaling pathways and ultimately influence the metabolism or growth vigor, RNA-Seq was conducted to evaluate the changed pathways in the triploid loquats. Correlation analyses showed that the three replicates were highly correlated (Figure S3). The density distribution of the gene expression levels was analyzed, and as is displayed in Figure S4A, most genes of the hybrids were expressed between the parents. The length of the obtained genes ranged from 200 nt to more than 3000 nt (Figure S4B). 

**Figure 5:**
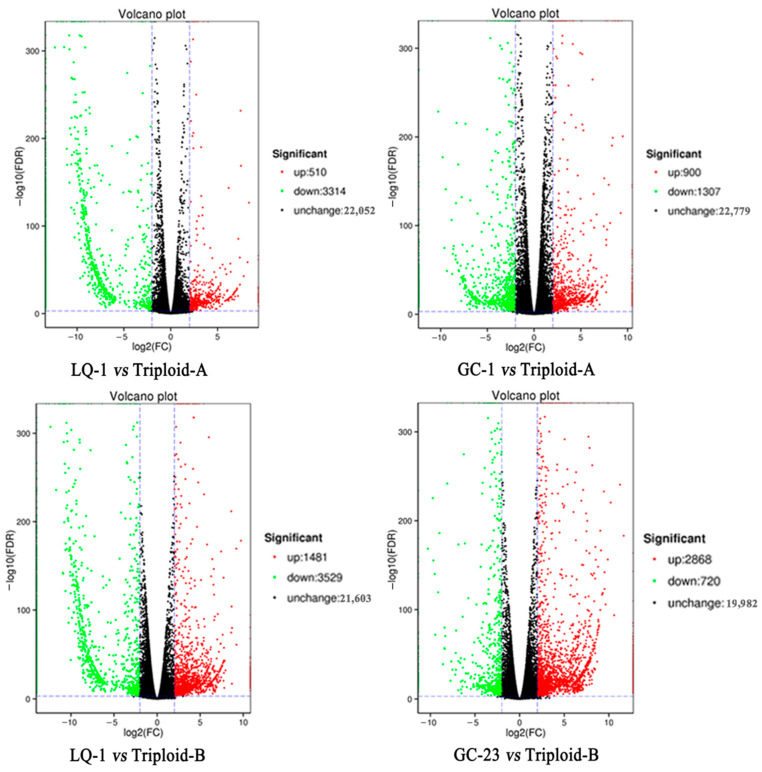
Volcano plots of the DEGs in the different comparisons between parent and hybrid.

To further investigate the differences between the pathways of the hybrids and of the parents, the DEGs screened based on the above selective criteria were further used to conduct Kyoto Encyclopedia of Genes and Genomes (KEGG) analyses. Totally, 6970 and 4384 DEGs were obtained between LQ-1 and GC-1/GC-23, respectively (Table 2), 6068 and 3910 of which were successfully annotated, and 2029 and 1279 of the annotated genes were mapped to the KEGG database (Table 2). Between LQ-1 and Triploid-A/Triploid-B, there were 3824 and 5010 DEGs, 1257 and 1553 of which were finally mapped to the KEGG database, respectively (Figure 5, Table 2). However, between GC-1 and Triploid-A or GC-23 and Triploid-B, totally, 407 and 978 DEGs were successfully mapped to the KEGG database, respectively (Figure 5, Table 2).

**Figure 6:**
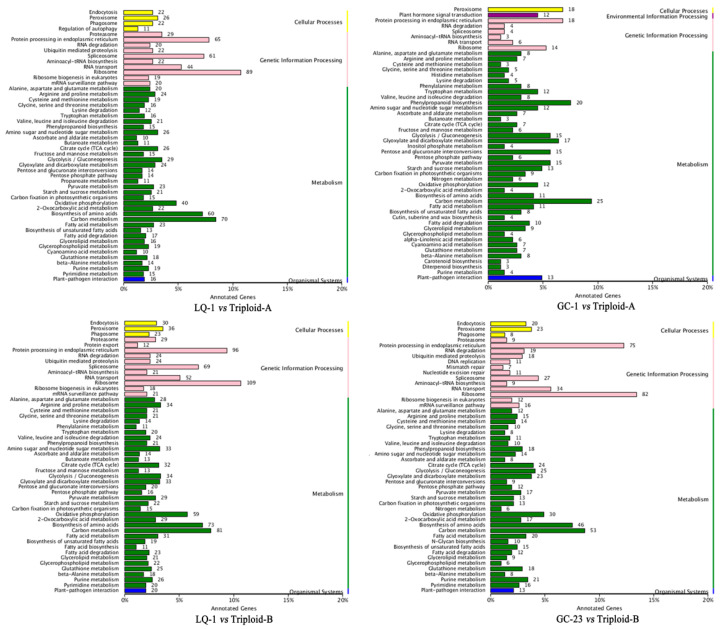
KEGG analyses of the DEGs in the different comparisons between the parent and hybrid.

The KEGG analyses suggested that significantly more DEGs were found to be associated with peroxisomes in the cellular process category in the comparisons of LQ-1 vs. Triploid-A and GC-1 vs. Triploid-A (Figure 6). Interestingly, the same results were found in the other cross combination (LQ-1 vs. Triploid-B and GC-23 vs. Triploid-B) (Figure 6). As reported in previous studies, apart from the functions involved in the fatty acid β-oxidation of peroxisomes that contributes to embryogenesis, seedling growth, etc., peroxisome activity was also closely correlated with photorespiration, which promotes photosynthetic efficiency [42,43]. This might further prove the high heterosis of the triploid hybrids in the cross combinations. Surprisingly, for the category of genetic information processing, protein-related functions, especially “Ribosome” and “Protein processing in endoplasmic reticulum”, were screened out based on different comparisons between the parents and hybrid groups, indicating that protein biosynthesis in the triploids might have been more exuberant than that in their parents (Figure 6). As we know, most enzymes are proteins, suggesting that the triploid loquats possibly demonstrate a higher metabolic level, which ultimately promotes the manifestation of heterosis. In addition, in the category of metabolism, the functions of “carbon metabolism” and “biosynthesis of amino acids” were found to play a dominant role in almost all the comparisons. Apart from these, “oxidative phosphorylation”, “glycolysis/gluconeogenesis”, “citrate cycle (TCA cycle)”, etc., also exhibited vigorous activity in the triploid loquats, which further suggested a stronger glyco-metabolism of the triploid hybrids (Figure 6). Collectively, the above results all pointed out that triploid loquats demonstrated stronger metabolic activity, such as a higher photosynthetic efficiency and a more robust carbon metabolism, and this confirmed the heterosis of triploid hybrids.

### 2.9. qRT-PCR Validation of Carbohydrate- and Protein-Related DEGs

**Figure 7:**
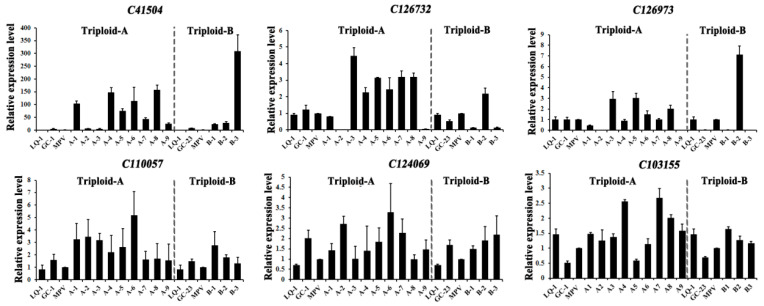
qRT-PCR validation of the growth-vigor-related DEGs, which were identified via RNA-Seq. Data are shown as means ± SD (*n* = 3). Error bars denote |S|D.

Our above KEGG analyses suggested that glyco-metabolism and protein biosynthesis might have greatly changed after the formation of triploid loquats since the photosynthetic efficiency, glycolysis/gluconeogenesis, functions related to genetic information processing, etc., were enriched. To further confirm these changes, we subsequently randomly selected several DEGs (*C41504*, *C126732*, *C126973*, *C110057*, *C124069*, and *C103155*) that belonged to the above categories (protein biosynthesis or glyco-metabolism), and the relative expression levels of these DEGs in the two cross lines were validated through qRT-PCR. For analytic convenience, the MPV value was adopted to determine the expression levels of these DEGs in the triploids; then, the expression of the DEGs was further divided into different five patterns: (a) MPL, whose gene expression level was in line with MPV; (b) HPL or LPL, whose gene expression level was in line with that of the high parent (HPL) or the low parent (LPL); (c) AHP or BLP, whose gene expression level was above the level of the high parent (AHP) or below the level of the low parent (BLP). Our results revealed that the expression of *C41504* and *C110057* demonstrated AHP in most of the triploids (A-1, A-4, A-5, A-6, A-7, A-8, A-9, B-1, B-2, B-3), exhibiting significant up-regulating tendencies (Figure 7, Table 3). For *C126732* and *C126973*, not only the AHP pattern but also the BLP pattern displayed obvious up-regulating or down-regulating tendencies (Figure 7, Table 3). Furthermore, apart from MPL and AHP, the expression of *C124069* and *C103155* showed the HPL trend in most of the triploids, and this was also consistent with the high-growth-vigor phenotypes in the hybrids (Figure 7, Table 3). Collectively, the above results suggested that the results obtained via RNA-Seq were accurate and believable, which covered the heterosis of the triploid loquats from other aspects.

## 3. Discussion 

We used ISSR and SSR markers to analyze the genetic diversity of the triploid loquats derived from the cross cultivation of loquat (Longquan-1 tetraploid) with two wild loquats (GC-1 and GC-23). In addition, RNA-Seq was further adopted to systematically illuminate the heterosis mechanisms of the triploid loquats. Finally, the genetic diversity of the triploid loquats was analyzed, and the relationships among the genotypes were addressed; furthermore, the heterosis mechanisms of the triploid loquats were analyzed. 

### 3.1. ISSR and SSR Markers Were Two Effective Molecular Markers for Loquat Breeding

ISSR and SSR marker systems prove to be useful tools to distinguish the genotypes, because a high level of polymorphic bands can be produced [44]. Both ISSR and SSR are widely used in plant breeding, e.g., for analyzing the genetic diversity of a population, for identifying the relationship of hybrids, QTL analyses, etc. [45,46,47,48]. Due to the fact that ISSRs are a universal marker system for species, they are widely used in loquat studies [49,50]. As for the SSR marker system, He et al. (2011) found that some apple SSRs could be successfully transferred to loquats, and finally, 39 apple SSRs were proved to be applicable in loquats [51]. Li et al. (2013) also applied apple EST-SSR primers to analyze the genetic diversity and relationships of 47 loquats and found that EST-SSR primers from apples were suitable for the analysis of loquat genetic diversity [52]. Therefore, in this study, we adopted the ISSR and SSR molecular markers to evaluate the genetic diversity of triploid loquats derived from hybridization. Surprisingly, both markers could be used to screen out the clear bands of triploid hybrids and their parents. In addition, a large number of polymorphic bands were identified using these two markers in triploid loquats compared with their parents. Although the average polymorphism ratio (19.32%) analyzed through SSRs was lower than that through ISSRs (43.75%), the two polymorphism ratios were higher than Wang’s (2008) [40]. However, it was worth noting that the variation trend of triploid loquats analyzed based on the two molecular markers was consistent. Thus, we thought that both ISSRs and SSRs could be used to effectively identify the genetic diversity of loquats. In order to confirm the correlation between ISSR and SSR markers, the Mantel test was conducted using the two similarity matrices (ISSR and SSR), and the result showed that the coefficient of correlation between ISSR and SSR was *r* = 0.848 (*p* < 0.01), which indicated that the results of the genetic diversity analyses through ISSRs and SSRs were credible (Figure 4).

### 3.2. Hybridization Was an Effective Way for Triploid Loquat Breeding

Due to the large amount of advantage traits that appear due to polyploidy, this further indicates that polyploids might possess a higher selective advantage over diploids [53,54]. Previous studies revealed that polyploids could be achieved via a variety of methods, including colchicine induction, somatic fusion, seed selection, and hybridization [54,55]. However, although somatic fusion technology provides an ideal route for polyploid generation, the technical limitations greatly restrict its application, which, until now, has only been successfully applied to citrus polyploid creation [56]. To date, hybridization is the optimal choice to create polyploids of different species due to the fact that a certain fertile germ cell can be produced by their parents. However, in this study, two groups of triploid lines were obtained by cross-fertilizing a tetraploid with diploids, and this was consistent with the theoretical analysis. Moreover, based on the law of the inheritance, the obtained triploid loquats surely contained two sets of chromosomes of the tetraploid parent (LQ-1) and one set of diploids (GC-1/GC-23); thus, the triploid hybrids might have been genetically closer to the tetraploid parent. Interestingly, our results in this study demonstrated that all the triploid hybrids showed closer relationships with the female parent in both the ISSR and SSR analyses, which was consistent with the above theoretical analysis and the results obtained by Wang (2008) [40]. Furthermore, the PCA analyses of ISSR and SSR markers also supported the above results (Figure 3).

### 3.3. The Formation of Triploid Loquats Was Accompanied by Extensive Genomic Variation

One of the most important characteristics of hybridization in plant breeding is that extensive genetic variation can be produced in the hybrids via sexual reproduction, which can further enrich the genetic diversity among the genotypes [57,58]. With a large number of genetic variations, some new traits may appear in the hybrids, which might exhibit more vigorous growth [59]. That is why genetic diversity is regarded as an important factor for breeding programs, fruit improvement, and germplasm management [60]. In addition, crossbreeding is also recognized as a unique driving force in the evolution process of the plant kingdom [61]. Studies on maize, wheat, *B. napus*, and rice found that there were a lot of genetic variations occurring in hybrids compared with their parents [62,63,64]. Generally, a suitable selection of hybrid parents is critically important for improving the variations in the hybrids, especially the genetic distance between the male and female parents [65]. That is to say, increasing the genetic distance between the parents to a limited extent is beneficial for the genetic variation frequency and for improving the heterosis of the hybrids [66]. However, loquats were found, in several studies, to have a narrow genetic distance, and genetic bottlenecks greatly limited their genetic variations. For example, in studies on the genetic diversity of cultivated loquats using isozymes and some molecular markers, such as amplified fragment length polymorphisms (AFLP), SSRs, and randomly amplified polymorphic DNA (RAPD), a high level of uniformity was found to exist among the cultivated loquats [52,67,68,69]. 

In this study, in order to broaden the genetic distance between the parents and increase the level of genetic variations in the hybrids, two wild loquats were selected as male parents, and we used two molecular marker systems (ISSRs and SSRs) to analyze the genetic variations in triploid loquats. The results of ISSRs showed that 43.75% of loci were polymorphic sites. Moreover, the 20 ISSR markers shared an average PIC value of 0.733; only 2 ISSR markers (ISSR-858 and ISSR-892) demonstrated a PIC value lower than 0.5. For SSRs, 19.32% of alleles were polymorphic sites, and only two SSRs exhibited a PIC value lower than 0.5. Wang (2008) used a cultivated tetraploid loquat (Jiefangzhong) for crossing with a cultivated diploid loquat (Hunanzaoshu), and finally, nine triploid loquats were obtained [40]. Then, their genetic diversity and their parents were analyzed using ISSR molecular markers, through which it was found that only 1.7% of sites were polymorphic sites [40]. Compared with Wang (2008), the polymorphism ratios (43.75% for ISSRs and 19.32% for SSRs) in this study were significantly higher than those in Wang’s (2008) (1.7%), which reminded us that choosing a suitable combination of parents can greatly help to broaden the level of variations in hybrids. On the other hand, the above results suggested that both ISSRs and SSRs could be used to effectively identify the genetic diversity of loquats.

### 3.4. Alterations in Photosynthetic Efficiency and Glyco-Metabolism Resulted in Heterosis Manifestation in Triploid Loquats

Logically, the gene expression level changes in triploid loquats might ultimately alter or affect the phenotypes. A study on the heterosis of Chinese cabbage found that heterosis was promoted by numerous up-regulated genes [70]. However, as we know, genes exert regulative functions via signaling pathways. In Arabidopsis, a variety of pathways were found to be closely associated with heterosis, such as the photosynthesis and auxin pathways, etc. [71]. For plants, especially horticultural plants, photosynthesis- and glyco-metabolism-related pathways might be critically important for heterosis manifestation, since they can directly affect biomass accumulation. For instance, a study on heterosis manifestation in cotton (*Gossypium hirsutum*) hybrid “Huaza Mian H318” uncovered that enhanced photosynthesis and carbohydrate metabolism facilitated heterosis in H318 during the seeding stage [72]. Similar results were also found in studies on heterosis in maize and *Brassica napus* [73,74]. Interestingly, in this study, results comparable to the outcomes discussed above were found through KEGG analyses, that is, significant DEGs were related to photosynthetic-efficiency- and glyco-metabolism-related pathways, such as peroxisomes, carbon metabolism, glycolysis/gluconeogenesis, and citrate cycle (TCA cycle). Additionally, several protein-biosynthesis-related pathways, such as “ribosome”, “protein processing in endoplasmic reticulum”, and “biosynthesis of amino acids”, were screened out through KEGG analyses. It is well known that most of the enzymes are proteins, and in the altered photosynthetic process and the glyco-metabolism process, the corresponding changes in enzymatic biosynthesis can well adapt to metabolic needs. Meanwhile, these photosynthetic changes and the glyco-metabolism process were also confirmed via our previous analyses of the contents of basic physiological indicators (SS, SP, CA, CB, and TC) (Table S2) as well as leaf morphology observations (Figure S2, Table S3). To this end, it could be concluded that the changes in the photosynthetic efficiency and glyco-metabolism of triploid loquats might play a critical role in regulating heterosis in triploid loquats. However, it should be mentioned that the regulation of heterosis manifestation in triploid loquats might be complicated, for instance, with regard to the regulation of the expression levels of the specific genes involved in the manifestation of heterosis, the nature of the interactions among different pathways during heterosis manifestation, etc. Thus, more studies need to be conducted in future research to deeply elucidate the heterosis mechanisms in triploid loquats. 

Collectively, based on the above descriptions, this study could provide more details for triploid loquat crossbreeding and offer an effective molecular marker system for loquat assisted breeding.

## 4. Materials and Methods

### 4.1. Plant Lines

The materials used in this study included three parents (one tetraploid female parent, Longquan-1 tetraploid, designated as LQ-1; and two diploid male parents, designated as GC-1/GC-23) and two sets of triploid loquat lines (designated as Triploid-A and Triploid-B) (Table 4). The first triploid line (Triploid-A) was generated by hybridizing LQ-1 with GC-1, and finally, 9 true triploid loquats were obtained, which were designated as A-1, A-2, A-3…A-9. The second triploid line (Triploid-B) was generated by hybridizing LQ-1 with GC-23, and finally, 3 true triploid loquats were obtained, which were designated as B-1, B-2, and B-3 (Table 4). The hybridizations were conducted in 2013, and the hybrids were planted in 2015. The two diploid male parents, GC-1 and GC-23, were two wild loquats collected from Guizhou Province, China, and were naturally grown in a rocky desertified region with a long genetic distance from cultivar loquat LQ-1. All the plants were grown at Experimental Base of the College of Horticulture and Landscape Architecture, Southwest University, China, under natural environmental conditions.

### 4.2. Ploidy Validation

The ploidy of the triploid hybrids obtained from the two combinations was validated through both chromosome preparation and flow cytometry analyses. The method for chromosome preparation was that proposed by Wen et al. (2020) [75]. Briefly, root-tip tissues with a length of about 1 cm were treated with 0.002 mol/L 8-hydroxyquinoline aqueous solution for 4 h and then fixed in Carnoy’s solution overnight. Subsequently, the apical meristems were cut into the size of about 1 mm^3^ for the following enzymolysis. Next, the enzyme-treated apical meristems were removed from Carnoy’s solution for final chromosome preparation on a slide. The chromosomes were stained with 5% Giemsa and visualized under a microscope (Olympus, Tokyo, Japan). Flow cytometry analyses were conducted as was described by Galbraith et al. (1983) [76]. The stain method used in this study was proposed by Miller et al. (2012) [77]. A BD Accuri C5 flow cytometer (BD Bioscience, San Jose, CA, USA) was adopted for the flow cytometry analyses in this study. 

### 4.3. DNA Extraction and Examination

Young and fresh leaves were powdered with liquid nitrogen, and their genomic DNA was extracted using the slightly modified CTAB (cetyltrimethyl ammonium bromide) method described by Liu et al. (2005) [78]. A Nanodrop 2000 spectrophotometer and 1% agarose gel were used to check the quality of the DNA.

### 4.4. ISSR and SSR Primers 

The ISSR primers used in the study were obtained from the ISSR primer database released by University of British Columbia (UBC), and 20 SSR primers were provided by Wu et al. (2015) [32]. All the primers were synthesized by Invitrogen^TM^ Life Technologies (Shanghai, China).

### 4.5. PCR and Electrophoreses 

All the DNA was adjusted to a final concentration of 20–40 ng/μL. For analyses of the polymerase chain reaction (PCR) of both ISSRs and SSRs, the final reaction mixes contained 1 μL of genomic template DNA, 0.5 U of *Taq* polymerase, 2 μL of 10 × PCR buffer, 2 μL of MgCl_2_ (25 mM), 0.5 μL of dNTP mixture (10 mM), and 0.5 μL of each forward and reverse primer (10 pmol/μL), with the addition of ddH_2_O to the final volume of 20 μL. *Taq* polymerase, 10 × PCR buffer, MgCl_2_, and dNTP mixture were bought from Takara Biotechnology Company (Dalian, China). PCR amplifications were performed on an Eppendorf master cycler (gradient; No. 5331-41264; Eppendorf AG, Hamburg, Germany) under the following conditions: 94 °C for 3 min and then 35 cycles at 94 °C for 30 s; annealing at the proper temperature for 30 s and at 72 °C for 30 s; then, a final step at 72 °C for 10 min. The annealing temperature of SSR primers was set as that described by Wu et al. (2015) [32], and ISSRs were set as those described in the ISSR primer database. The PCR products of ISSRs were detected using 1.5% agarose gel, and the SSR products were separated via 8% polyacrylamide gels and stained with silver [79].

### 4.6. MRNA-Seq Analyses of the Leaf Tissues in Different Ploidy Loquats

RNA was isolated from the fresh leaves of different ploidy loquats using an Aidlab RNA extraction kit (Aidlab Biotechnologies, Beijing, China). The concentration of RNA was detected using Agilent 2100 (Agilent technologies, Inc., Santa Clara, CA, USA); subsequently, total RNA was subjected to rRNA depletion and cDNA library construction. The cDNA synthesis and library construction were performed following the manufacturer’s instructions (Biomarker Technologies, Beijing, China). Finally, mRNA-Seq was conducted on the Illumina HiSeq4000 platform. In this study, the RNA samples in the two cross combinations were mixed at an equal concentration to form a composite sample, respectively; finally, two composite samples, named Triploid-A and Triploid-B, were obtained for RNA-Seq analyses. Three biological replicates were sequenced for each phenotype. 

After sequencing, the adapters were removed from the raw reads, which were further processed based on the following two criteria: (a) reads with more than 10% of vague bases and (b) reads with a length that was shorter than 20 nt were eliminated. Subsequently, the clean reads obtained were mapped to the loquat genome, which was de novo assembled by Biomarker Technologies. In addition, the expression level of the mapped genes was quantified based on the FPKM (fragments per kilobase of transcript per million mapped reads) values, and the differentially expressed genes (DEGs) were determined using the “base mean”, which was acquired via the DEGeq package. Finally, the significant difference in genes between two different samples was identified using the following two criteria: (a) false-discovery rate (FDR) ≤ 0.01 and (b) |log2 FPKM| ≥ 2. 

### 4.7. Quantitative Real-Time PCR Validation

The fresh leaves from parents and triploid hybrids were collected, and RNA was isolated; then, cDNA was synthesized. The methods for RNA isolation and cDNA synthesis were the same as those for RNA-Seq analyses. SYBR-based qRT-PCR was performed on Step-One Real-Time System (Gene Company Limited, Hong Kong, China) as follows: pre-denaturation for 10 s at 95 °C followed by 40 cycles at 95 °C for 5 s and 62 °C for 10 s. All the samples were performed in triplicates, and the relative expression levels were determined using Microsoft Excel 2010 through the delta–delta Ct method. The *Actin* gene, which was selected by our laboratory, was adopted as an internal control. The primers used for qRT-PCR analyses in this study are listed in Table S1.

### 4.8. Data Analysis

Mid-parent value (MPV) and mid-parent heterosis (MPH) were adopted to evaluate the degree of triploid heterosis. These two indexes (MPV and MPH) were calculated according to a report by Turner (1953) [80]. Briefly, MPV was calculated based on the genomic contributions of parents to hybrids, i.e., MPV = 2/3 LQ-1 + 1/3 GC-1/23; MPH was calculated using the formula MPH = (triploids − MPV)/MPV × 100%. For the ISSR and SSR loci, bands were manually scored into binary data using the method described by Liu et al. (2018) [81]. Briefly, loci were scored as “1” if the bands appeared and “0” if the loci were without bands. All the parameters in this study were obtained from the ISSR and SSR markers: observed number of alleles (*N*_Aobs_); number of alleles observed by Wu et al. (2015) (*N*_A_ Wu) [32]; Shannon index (*I*) and expected heterozygosity (*H*_e_ = 1 − ∑*p_i_*^2^, where *p_i_* is the frequency of the *i*th allele), which were calculated using POPGENE software (version 1.31, created by Yeh et al. Edmonton, Canada) [82]; and observed heterozygosity (*H*_o_, calculated as the number of heterozygous genotypes divided by the total number of genotypes), Wright’s fixation index (*F* = 1 − *H*_o_/*H*_e_) [83], and polymorphism information content (PIC), which were calculated using PowerMarker v3.25 (North Carolina State, USA) (Nei 1973) [82]. Then, two (ISSR and SSR) similarity matrices were generated using Nei’s genetic distance based on the binary data [84]. The two similarity matrices were further used for dendrogram construction using the unweighted pair-group method (UPGMA) cluster analysis using NTSYSpc 2.10e software (Applied Biostatistics Inc., New York, NY, USA), together with principal component analyses (PCAs) and the Mantel test [85,86]. Genetic variation patterns were mainly associated with two types of bands: gain bands (a novel band that no parent had) and loss bands (a band presented in both parents that disappeared in the hybrids) [87,88]. Thus, the two patterns of genetic variations, gain bands and loss bands were adopted for the following analyses.

## Figures and Tables

**Table 1:** List of the genetics parameters of the 20 ISSR and 20 SSR primers of the two cross lines.

Locus Name	TB	PB	PPB			*I*	*H*e	*H*o	*F*	PIC
ISSR										
ISSR-807	6	2	33.33%				-^a^	-^a^		0.810
ISSR-811	6	0	0.00%				-^a^	-^a^		0.810
ISSR-823	4	2	50.00%				-^a^	-^a^		0.703
ISSR-825	7	4	57.14%				-^a^	-^a^		0.818
ISSR-834	7	4	57.14%				-^a^	-^a^		0.811
ISSR-835	9	4	44.44%				-^a^	-^a^		0.858
ISSR-836	7	2	28.57%				-^a^	-^a^		0.835
ISSR-840	6	3	50.00%				-^a^	-^a^		0.809
ISSR-845	6	4	66.67%				-^a^	-^a^		0.780
ISSR-853	9	6	66.67%				-^a^	-^a^		0.828
ISSR-858	2	0	0.00%				-^a^	-^a^		0.375
ISSR-874	16	4	25.00%				-^a^	-^a^		0.930
ISSR-876	7	3	42.86%				-^a^	-^a^		0.839
ISSR-879	6	4	66.67%				-^a^	-^a^		0.810
ISSR-880	5	2	40.00%				-^a^	-^a^		0.737
ISSR-886	5	0	0.00%				-^a^	-^a^		0.768
ISSR-887	5	2	40.00%				-^a^	-^a^		0.755
ISSR-892	1	0	0.00%				-^a^	-^a^		monomorphic
ISSR-899	6	5	83.33%				-^a^	-^a^		0.562
ISSR-900	8	5	62.50%				-^a^	-^a^		0.813
Mean	6.4	2.8	43.75%							0.733
Total	128	56	43.75%							
Locus name				*N*_Aobs_	*N*_A_ Wu	*I*	*H*e	*H*o	*F*	PIC
SSR										
ssrEJ005	5	2	40.00%	5	2	0.693	0.524	0.273	0.479	0.822
ssrEJ012	4	0	0.00%	4	5	-^a^	-^a^	-^a^	-a	0.634
ssrEJ014	4	0	0.00%	4	3	0.185	0.091	0.091	0	0.525
ssrEJ037	4	1	25.00%	4	3	0.185	0.091	0.091	0	0.740
ssrEJ039	5	3	60.00%	5	2	0.185	0.091	0.091	0	0.678
ssrEJ042	4	0	0.00%	4	3	0.656	0.485	-^a^	-a	0.674
ssrEJ046	6	0	0.00%	6	6	0.690	0.520	0.909	-0.748	0.810
ssrEJ049	6	0	0.00%	6	5	0.689	0.520	0.909	-0.748	0.819
ssrEJ061	4	0	0.00%	4	6	-^a^	-^a^	-^a^	-a	0.457
ssrEJ066	4	0	0.00%	4	4	-^a^	-^a^	-^a^	-a	0.634
ssrEJ075a	6	0	0.00%	6	3	-^a^	-^a^	-^a^	-a	0.697
ssrEJ086	3	1	33.33%	3	3	0.305	0.173	-^a^	-a	0.553
ssrEJ088	4	0	0.00%	4	4	0.305	0.173	-^a^	-a	0.752
ssrEJ095b	4	0	0.00%	4	4	0.693	0.524	0.818	-0.561	0.701
ssrEJ104	4	0	0.00%	4	4	-^a^	-^a^	-^a^	-a	0.702
ssrEJ106	1	0	0.00%	1	2	-^a^	-^a^	-^a^	-a	monomorphic
ssrEJ271	5	3	60.00%	5	6	0.185	0.091	0.091	0	0.743
ssrEJ282	6	3	50.00%	6	5	0.398	0.247	0.273	-0.105	0.872
ssrEJ324	4	1	25.00%	4	6	0.689	0.520	0.909	-0.748	0.670
ssrEJ329b	5	3	60.00%	5	3	0.185	0.091	0.091	0	0.766
Mean	4.4	0.85	19.32%	4	4	0.302	0.207	0.227	-0.122	0.662
Total	88	17	19.32%	88	79					

Note: TB, total bands; PB, polymorphic bands; PPB, percentage of polymorphic bands. ^a^ not determined. *N*_Aobs_, observed number of alleles; *N*_A_ Wu, number of alleles observed by Wu et al. (2015); *I*, Shannon index; *H*e, expected heterozygosity; *H*o, observed heterozygosity; *F*, Wright’s fixation index; PIC, polymorphic information content value.

**Table 2:** Characteristics of the differentially expressed genes generated from the transcriptome analyses of the different comparative groups.

Comparison between Different Groups	Total DEGs	Number of the Annotated DEGs	Percentage ^a^ (%)	Number of KEGG-Mapped DEGs	Percentage ^b^ (%)
LQ-1 vs. GC-1	6970	6068	87.06	2029	29.11
LQ-1 vs. Triploid-A	3824	3619	94.64	1257	32.87
GC-1 vs. Triploid-A	2207	1799	81.51	407	18.44
LQ-1 vs. GC-23	4384	3910	89.19	1279	29.17
LQ-1 vs. Triploid-B	5010	4608	91.98	1553	31.00
GC-23 vs. Triploid-B	3588	2855	79.57	978	27.26

^a^ percentage of the annotated DEGs; ^b^ percentage of DEGs mapped to the KEGG database (https://www.kegg.jp/kegg/, accessed on 20 August 2022).

**Table 3:** The expression patterns of the selected DEGs in triploid loquat.

	MPL ^a^	HPL ^b^	LPL ^c^	AHP ^d^	BLP ^e^
*C41504*	N/A	A-2, A-3	N/A	A-1, A-4, A-5, A-6, A-7, A-8, A-9, B-1, B-2, B-3	N/A
*C126732*	N/A	N/A	N/A	A-3, A-4, A-5, A-6, A-7, A-8, B-2	A-1, A-2, A-9, B-1, B-3
*C126973*	A-4, A-7	N/A	N/A	A-3, A-5, A-6, A-8, B-2	A-1, A-2, A-9, B-1, B-3
*C110057*	N/A	A-7, A-8, A-9, B-3	N/A	A-1, A-2, A-3, A-4, A-5, A-6, B-1, B-2	N/A
*C124069*	A-3, A-8	A-1, A-4, A-5, A-7, A-9, B-1, B-2	N/A	A-2, A-6, B-3	N/A
*C103155*	A-6, B-3	A-1, A-2, A-3, A-9, B-1, B-2	A-5	A-4, A-7, A-8	N/A

^a^: Gene expression level in line with the mid-parent value (MPV); ^b^: Gene expression level in line with that of the high parent; ^c^: Gene expression level in line with that of the low parent; ^d^: Gene expression level above the level of the high parent; ^e^: Gene expression level below the level of the low parent.

**Table 4:** The loquat materials in the two cross combinations used in this study.

Cross Combinations	Serial Number of Triploid Loquats (Triploid-A)								
LQ-1× GC-1	A-1	A-2	A-3	A-4	A-5	A-6	A-7	A-8	A-9
	Serial Number of Triploid Loquats (Triploid-B)								
LQ-1 × GC-23	B-1	B-2	B-3						

## Data Availability

The datasets generated and analyzed during the present study are available from the corresponding author upon reasonable request.

## Supplementary Materials

The following supporting information can be downloaded at: https://www.mdpi.com/article/10.3390/ijms231911337/s1.
