# Supplementary material for: Photosynthetic Efficiency and Glyco-Metabolism Changes in Artificial Triploid Loquats Contribute to Heterosis Manifestation
Source: Int J Mol Sci. 2022 Sep 26;23(19):11337. doi: 10.3390/ijms231911337 (PMC9570370; doi:10.3390/ijms231911337)

Table S1. Primers used for the qRT-PCR analyses in this study.

Primers	Sequence (5'-3')	Tm	Functions of the targets
C41504-F	atggttgttgagaatggcggt	60 °C	Leaf growth related gene
C41504-R	agcttgtctgtgatccagggc	60 °C	Leaf growth related gene
C126732-F	aaattgctgctctgatctctct	60 °C	Sucrose/Starch metabolic related gene
C126732-R	gaccttgtagtctcttccctcg	60 °C	Sucrose/Starch metabolic related gene
C126973-F	ttggggacggagtagacatag	60 °C	Sucrose/Starch metabolic related gene
C126973-R	tcgtggagaggaactgaaggg	60 °C	Sucrose/Starch metabolic related gene
C110057-F	gaggctaccttgactacc	60 °C	Sucrose/Starch metabolic related gene
C110057-R	aaaatgagaaaaccgacc	60 °C	Sucrose/Starch metabolic related gene
C124069-F	tatccgcagaggagaattccgcaag	60 °C	Sucrose/Starch metabolic related gene
C124069-R	ccaccaaaccaaggtatgtaccagg	60 °C	Sucrose/Starch metabolic related gene
C103155-F	ccgcttcaggagatggagac	60 °C	Protein processing in endoplasmic reticulum
C103155-R	cgacaatgccaatgtaggag	60 °C	Protein processing in endoplasmic reticulum
Actin-F	atccttcgtctggaccttgc	60 °C	Reference gene
Actin-R	gacaatttcccgttcagcagt	60 °C	Reference gene

Table S2. The five physiological indicator examinations of the parents and F1 hybrids (Triploid-A, Triploid-B), and Mid-parent Heterosis (MPH) of the F1 hybrids (Triploid-A, Triploid-B).

	Soluble starch (SS)	Soluble protein (SP)	Chlorophyll A (CA)	Chlorophyll B (CB)	Total Chlorophyll (TC)
LQ-1	0.23±0.0054[a]	148.57±0.0694	0.8778±0.0235	0.5697±0.0109	1.4474±0.0343
GC-1	0.17±0.0119	146.33±0.0694	0.9944±0.0183	0.7005±0.0247	1.6949±0.0430
MPV[b]-A	0.21±0.0051	147.82±0.0231	0.9167±0.0218	0.6133±0.0153	1.5299±0.0371
A-1	0.23±0.0025<br>10.82%[c]+0.79%	150.29±0.0694<br>1.67%+1.16%	0.9710±0.0131<br>5.93%+(-2.36%)	0.6982±0.0166<br>13.84%+(-0.33%)	1.6692±0.0291<br>9.10%+(-1.52%)
A-2	0.22±0.0250<br>2.80%+(-6.51%)	147.65±0.0694<br>-0.12%+(-0.62%)	0.9283±0.0232<br>1.27%+(-6.65%)	0.6102±0.0280<br>-0.50%+(-12.89%)	1.5385±0.0213<br>0.56%+(-9.23%)
A-3	0.31±0.0227<br>46.18%+32.95%	138.56±0.5236<br>-6.27%+(-6.74%)	0.9625±0.0227<br>5.00%+(-3.21%)	0.6141±0.0290<br>0.13%+(-12.34%)	1.5765±0.0517<br>3.04%+(-6.99%)
A-4	0.26±0.0238<br>23.84%+12.63%	149.53±0.4548<br>1.16%+0.65%	0.8833±0.0154<br>-3.64%+(-11.18%)	0.5574±0.0116<br>-9.11%+(-20.42%)	1.4407±0.0267<br>-5.83%+(-15.00%)
A-5	0.23±0.0038<br>11.98%+1.84%	149.93±0.1387<br>1.43%+0.92%	1.0983±0.0120<br>19.81%+10.44%	0.8182±0.0258<br>33.40%+16.80%	1.9164±0.0196<br>25.26%+13.07%
A-6	0.24±0.0110<br>13.42%+3.16%	138.84±2.0468<br>-6.08%+(-6.55%)	1.0665±0.0234<br>16.35%+7.25%	0.7762±0.0490<br>26.56%+10.80%	1.8427±0.0723<br>20.44%+8.72%
A-7	0.18±0.0140<br>-16.00%+(-23.61%)	133.07±0.3670<br>-9.98%+(-10.43%)	1.0915±0.0155<br>19.07%+9.76%	0.8155±0.0310<br>32.98%+16.42%	1.9070±0.0465<br>24.65%+12.51%
A-8	0.24±0.0040<br>14.36%+4.01%	128.59±0.7307<br>-13.01%+(-13.45%)	0.9742±0.0340<br>6.28%+(-2.04%)	0.6298±0.0350<br>2.70%+(-10.09%)	1.6040±0.0690<br>4.84%+(-5.36%)
A-9	0.24±0.0260<br>14.73%%+4.34%	124.02±0.8409<br>-16.10%+(-16.52%)	0.9619±0.0173<br>4.94%+(-3.27%)	0.6409±0.0187<br>4.50%+(-8.51%)	1.6028±0.0359<br>4.77%+(-5.43%)
LQ-1	0.23±0.0054[a]	148.57±0.0694	0.8778±0.0235	0.5697±0.0109	1.4474±0.0343
GC-23	0.33±0.0197	137.92±0.6616	0.8736±0.0101	0.5157±0.0091	1.3893±0.0177
MPV[b]-B	0.26±0.0058	145.02±0.2666	0.8764±0.0189	0.5517±0.0087	1.4281±0.0276
B-1	0.48±0.0401<br>83.53%[c]+47.41%	137.68±0.8855<br>-5.06%+(-7.33%)	1.0722±0.0201<br>22.35%+22.15%	0.7675±0.0390<br>39.12%+34.72%	1.8397±0.0590<br>28.83%+27.10%
B-2	0.24±0.0193<br>-7.46%+(-25.68%)	145.56±0.5548<br>0.38%+(-2.02%)	0.9433±0.0159<br>7.64%+7.47%	0.5959±0.0194<br>8.01%+4.60%	1.5392±0.0351<br>7.79%+6.34%
B-3	0.69±0.0125<br>164.09%+112.11%	143.76±0.2501<br>-0.87%+(-3.23%)	0.7829±0.0266<br>-10.66%+(-10.80%)	0.4754±0.0199<br>-13.84%+(-16.56%)	1.2583±0.0465<br>-11.89%+(-13.07%)

[a] mean ± standard deviation; [b] Mid-parent value (MPV) was calculated based on the genomics contribution by the two parents, *i.e.,* 2/3 LQ-1+1/3 GC-1/GC-23;

[c]Mid-parent heterosis (MPH) was calculated by using the formula MPH = (triploids-MPV)/MPV*100%; This table was cited and reproduced from Liu et al., (2019) [37].

Table S3. Leaf thickness analyses of the parents and F1 hybrids (Triploid-A, Triploid-B), and Mid-parent Heterosis (MPH) of the F1 hybrids (Triploid-A, Triploid-B).

Categories	LQ-1	GC-1	GC-23	A-1	A-2	A-3	A-4	A-5	A-6	A-7	A-8	A-9	B-1	B-2	B-3
Leaf Thickness (mm)	0.28 (0.02)*	0.16 (0.01)	0.22 (0.02)	0.27 (0.01)	0.27 (0.01)	0.31 (0.02)	0.30 (0.02)	0.38 (0.01)	0.35 (0.01)	0.28 (0.01)	0.28 (0.01)	0.30 (0.01)	0.43 (0.02)	0.35 (0.01)	0.40 (0.02)
MPV-A[a]: 0.24 (0.01); MPV-B[a]: 0.26 (0.01)															
MPH[b]				14.29%	14.46%	30.83%	25.16%	60.73%	45.66%	16.90%	16.85%	26.43%	65.66%	35.95%	54.73%

* mean ± standard deviation; [a] Mid-parent value (MPV) was calculated based on the genomics contribution by the two parents, *i.e.*, 2/3 LQ-1+1/3 GC-1/GC-23; [b]Mid-parent heterosis (MPH) was calculated by using the formula MPH = (triploids-MPV)/MPV*100%; This table was cited and reproduced from Liu et al., (2019) [37].

Figure S1. Ploidy validation of the triploid loquats in the two cross combinations. (A) the representative images of the chromosome count and the obtained seeds in the two combinations. (B-D) ploidy validation of the triploid hybrids using flow cytometry. This figure was reproduced from Liu et al. (2018) [47].

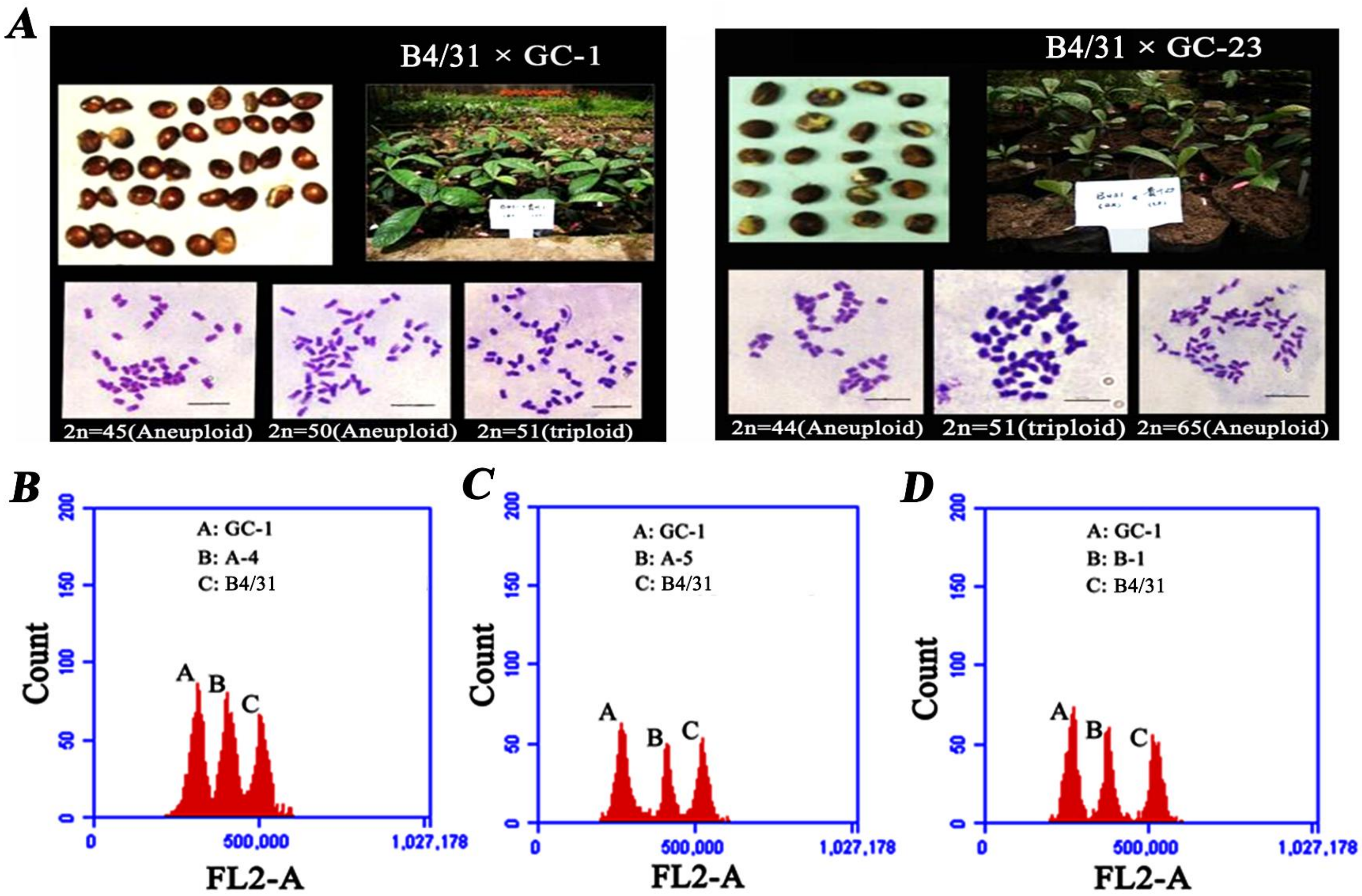

Figure S2. Leaf micro-structure observations of the triploid hybrids and the parents. All the sub-picture were scaled down at the same proportion. The size of the bar showed in the picture was 50 μm. This figure was cited from Liu et al., (2019) [37].

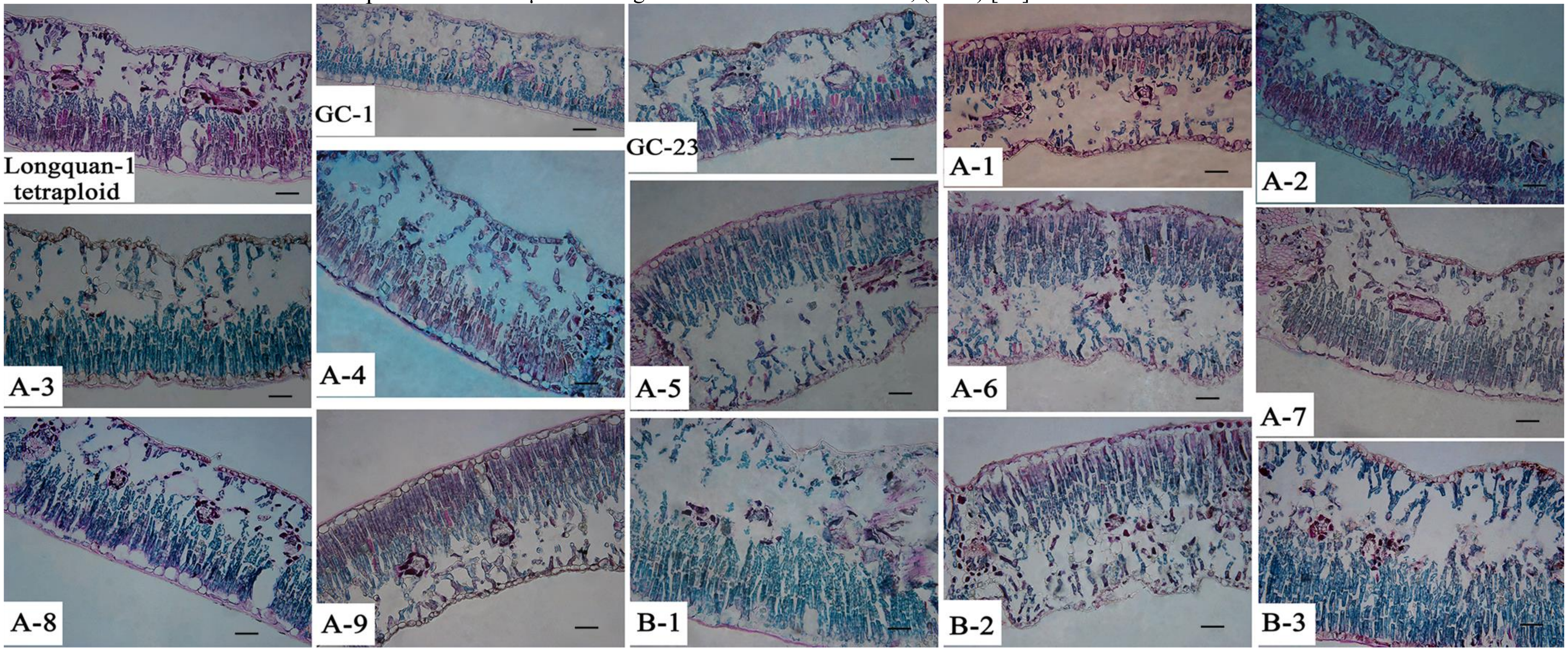

Figure S3. Pearson's correlation coefficient of different replicates in the transcriptome analysis. The correlation coefficient was calculated by using log10 (FPKM+1).

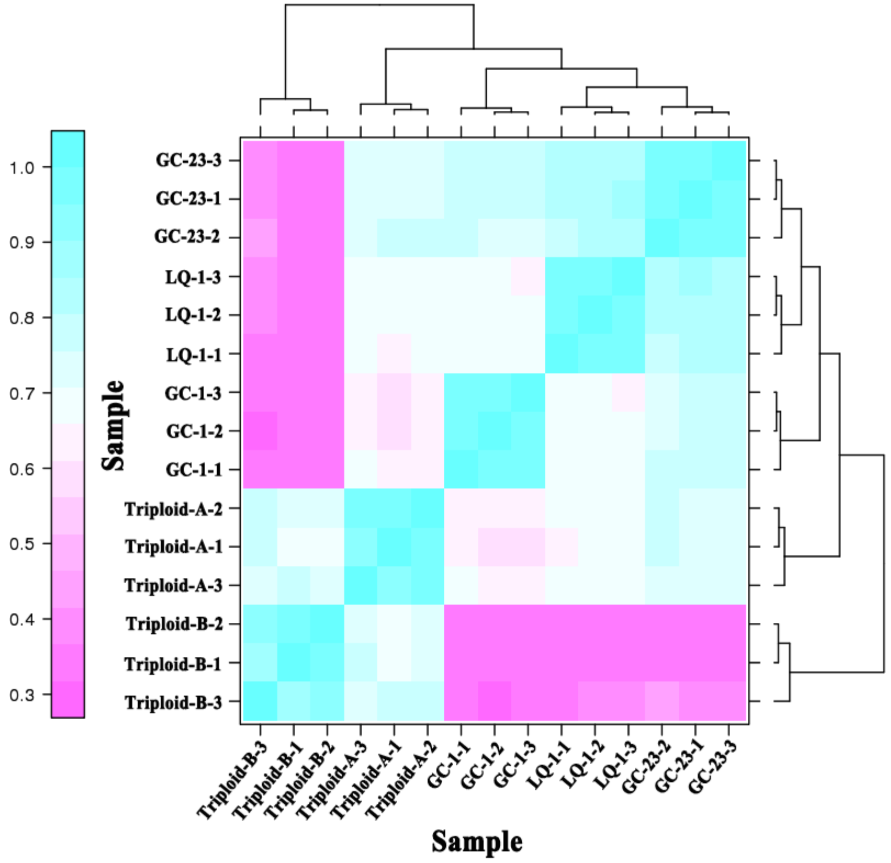

Figure S4. The expression (A) and length distributions (B) of the unigenes in each sample.

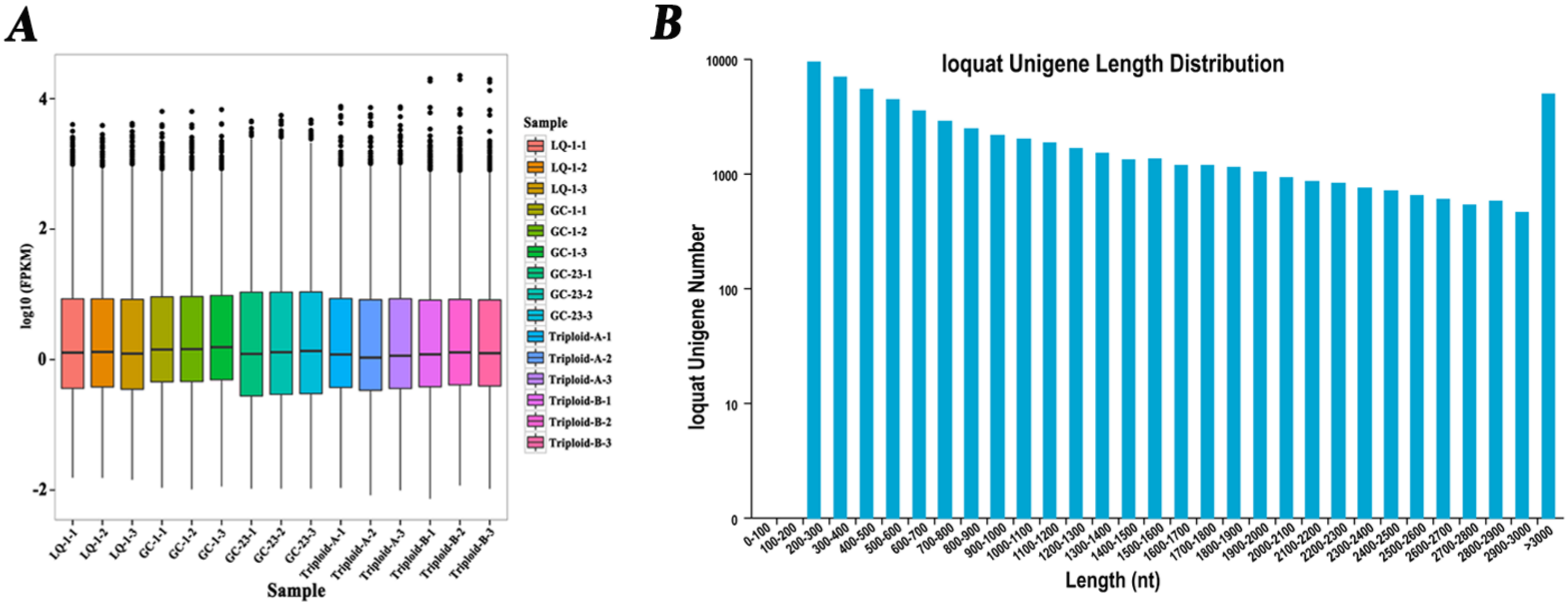

Supplement: Supplementary file 1 [file ijms-23-11337-s001.zip › ijms-1921925-supplementary.pdf]
